# Advantages of Arthroscopic Transosseous Suture Repair of the Rotator Cuff without the Use of Anchors

**DOI:** 10.1007/s11999-013-3148-7

**Published:** 2013-07-09

**Authors:** Shigehito Kuroda, Noriyuki Ishige, Motohiko Mikasa

**Affiliations:** Shoulder Division of the Clinical Research Center, Matsudo Orthopaedic Hospital, 1-161 Asahi-cho, Matsudo City, Chiba Prefecture 271-0043 Japan

## Abstract

**Background:**

Although arthroscopic anchor suturing is commonly used for rotator cuff repair and achieves good results, certain shortcomings remain, including difficulty with reoperation in cases of retear, anchor dislodgement, knot impingement, and financial cost. In 2005, we developed an anchorless technique for arthroscopic transosseous suture rotator cuff repair.

**Description of Technique:**

After acromioplasty and adequate footprint decortication, three K-wires with perforated tips are inserted through the inferior margin of the greater tuberosity into the medial edge of the footprint using a customized aiming guide. After pulling the rotator cuff stump laterally with a grasper, three K-wires are threaded through the rotator cuff and skin. Thereafter, five Number 2 polyester sutures are passed through three bone tunnels using the perforated tips of the K-wires. The surgery is completed by inserting two pairs of mattress sutures and three bridging sutures.

**Methods:**

We investigated the retear rate (based on MR images at least 1 year after the procedure), total score on the UCLA Shoulder Rating Scale, axillary nerve preservation, and issues concerning bone tunnels with this technique in 384 shoulders in 380 patients (174 women [175 shoulders] and 206 men [209 shoulders]). Minimum followup was 2 years (mean, 3.3 years; range, 2–7 years). Complete followup was achieved by 380 patients (384 of 475 [81%] of the procedures performed during the period in question). The remaining 91 patients (91 shoulders) do not have 1-year postsurgical MR images, 2-year UCLA evaluation or intraoperative tear measurement, or they have previous fracture, retear of the rotator cuff, preoperative cervical radiculopathy or axillary nerve palsy, or were lost to followup.

**Results:**

Retears occurred in 24 patients (24 shoulders) (6%). The mean overall UCLA score improved from a preoperative mean of 19.1 to a score of 32.7 at last followup (maximum possible score 35, higher scores being better). Postoperative EMG and clinical examination showed no axillary nerve palsies. Bone tunnel-related issues were encountered in only one shoulder.

**Conclusions:**

Our technique has the following advantages: (1) reoperation is easy in patients with retears; (2) surgical materials used are inexpensive polyester sutures; and (3) no knots are tied onto the rotator cuff. This low-cost method achieves a low retear rate and few bone tunnel problems, the mean postoperative UCLA score being comparable to that obtained by using an arthroscopic anchor suture technique.

**Level of Evidence:**

Level IV, therapeutic study. See Instructions for Authors for a complete description of levels of evidence.

**Electronic supplementary material:**

The online version of this article (doi:10.1007/s11999-013-3148-7) contains supplementary material, which is available to authorized users.

## Introduction

Arthroscopic rotator cuff repair using anchors is a common surgical approach that achieves good results. Recently, there have been changes in anchor suture techniques, from single row to double row and then to suture bridge. However, these procedures have certain limitations: (1) when a retear occurs, reoperation is difficult because the anchors are attached to the greater tuberosity; (2) suture anchors are expensive; (3) dislodgement of the anchor sometimes occurs [3]; and (4) knot impingement occasionally occurs, this being attributable to knots placed on the rotator cuff [16].

In an attempt to overcome these limitations, we have developed a technique for arthroscopic transosseous suture repair of the rotator cuff without the use of anchors, which has been used at our hospital since April 2005. Because only sutures are used in this procedure, problems resulting from the above-mentioned anchor suture technique do not occur. The only surgical materials used are inexpensive polyester sutures. Furthermore, as knots are not tied onto the rotator cuff but rather to the lower margin of the greater tuberosity, this technique avoids knot impingement.

We describe the details of this procedure and report its outcomes in terms of (1) retear rate, (2) total score on the UCLA Shoulder Rating Scale [6], (3) axillary nerve injury, and (4) issues concerning bone tunnels, such as breakage by a suture.

## Surgical Technique

**Fig. 1A–B:**
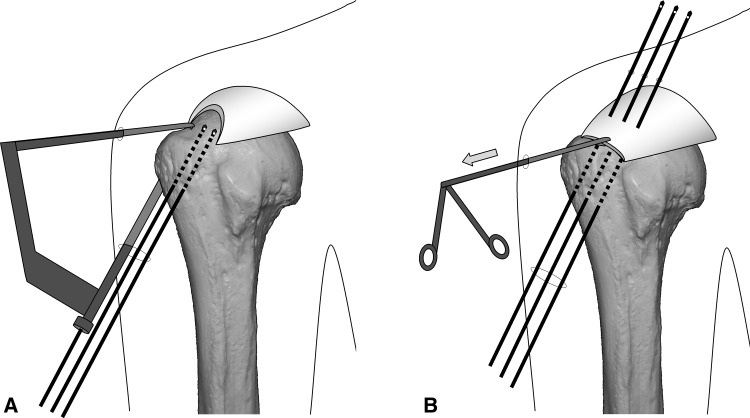
(**A**) The aiming tip of the drill guide passing through the anterolateral portal was placed on the medial edge of the footprint and three K-wires with perforated tips were inserted through the inferior margin of the greater tuberosity. (**B**) The rotator cuff stump was pulled laterally, and the K-wires were threaded through the rotator cuff and skin posterior to the acromioclavicular joint.

**Fig. 2A–B:**
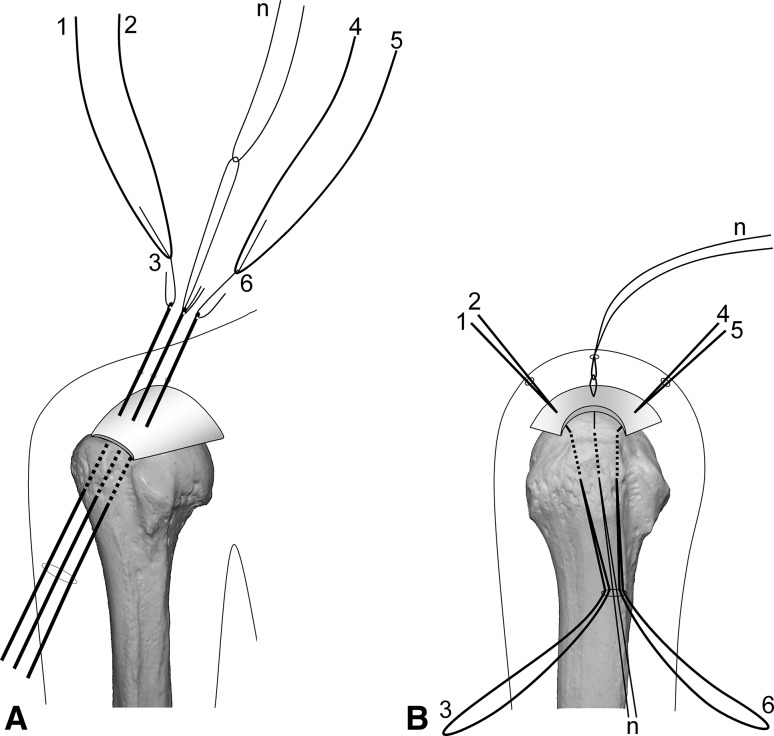
(**A**) Nylon threads tied with a single knot in the center of a 135-cm Number 2 polyester suture were passed through the perforated tips of the anterior and posterior K-wires. Nylon loops connected doubly in series were pulled through the central K-wire. These nylon loops are used for a relay for passing the mattress and central bridging sutures back and forth through the central bone tunnel. To clarify suture management, suture ends are numbered 1 to 8. (**B**) The two polyester loops (1-3-2, 4-6-5) and nylon loops (n) passing through the K-wires were pulled out through the lower portal.

**Fig. 3:**
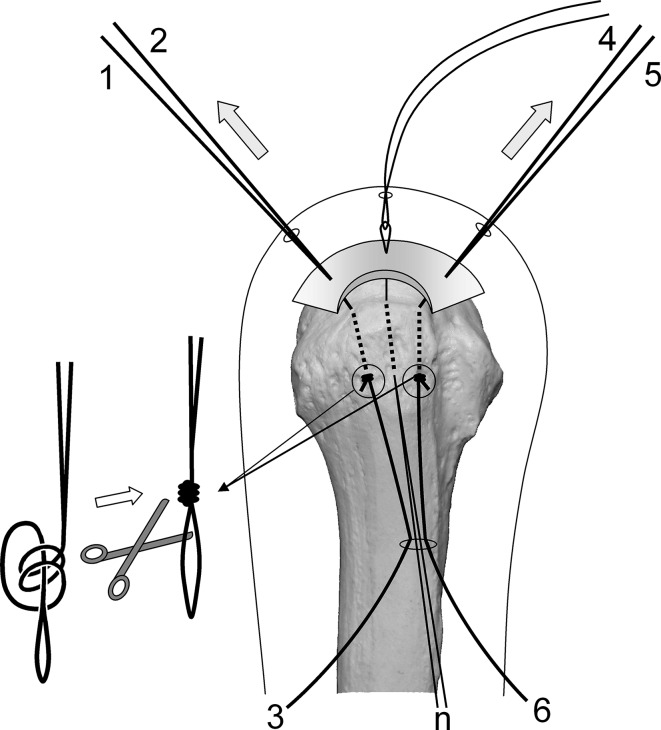
The anterior (4-6-5) and posterior (1-3-2) polyester loops pulled out were knotted, and one end of each loop was cut and pulled superiorly. The main knot then was sited at the inferior margin of the greater tuberosity.

**Fig. 4A–B:**
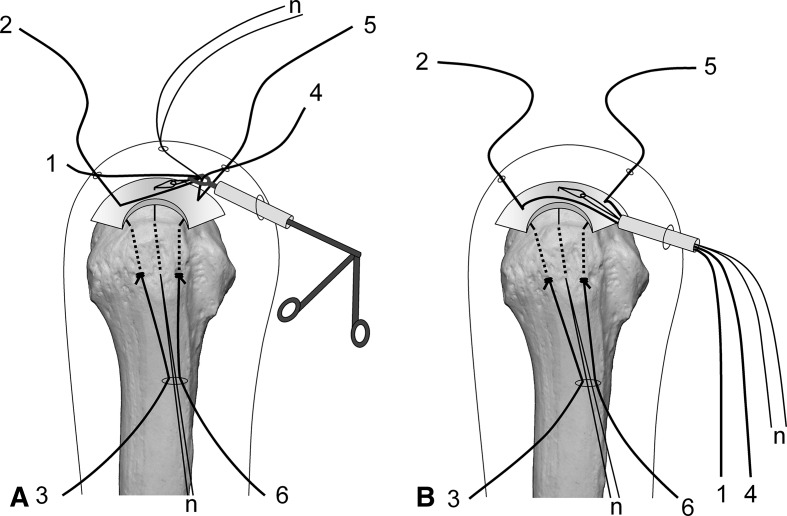
The central nylon relay thread (n) and one limb of each of the anterior (4) and posterior (1) polyester sutures inserted through the rotator cuff were (**A**) hooked and (**B**) pulled out through the anterolateral portal.

**Fig. 5A–B:**
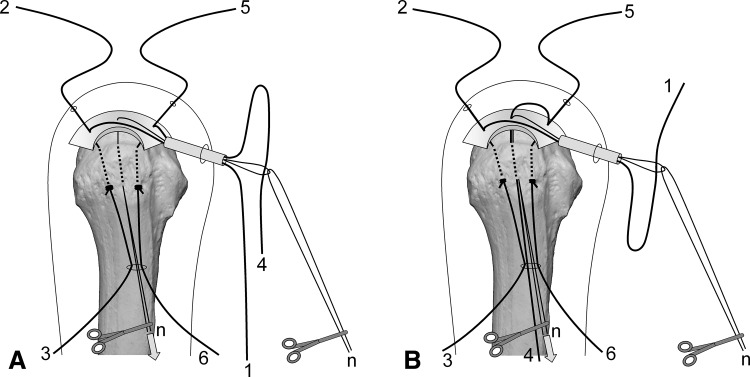
Each of the two polyester limbs that had been extracted through the anterolateral portal [(**A**) 4 and (**B**) 1] then was drawn separately through the lower portal passing through the central bone tunnel using the nylon loop.

**Fig. 6A–B:**
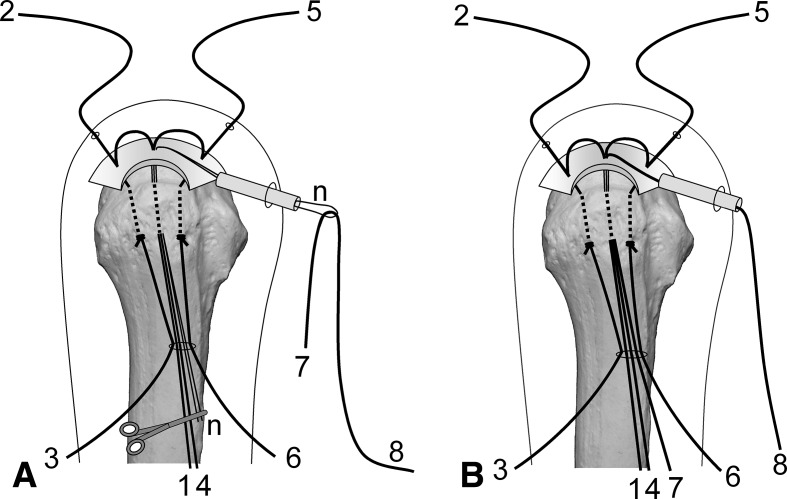
A 65-cm Number 2 polyester suture for central bridging (7-8) subsequently was (**A**) inserted into the nylon loop and (**B**) drawn through the central bone tunnel and lower portal by the loop.

**Fig. 7:**
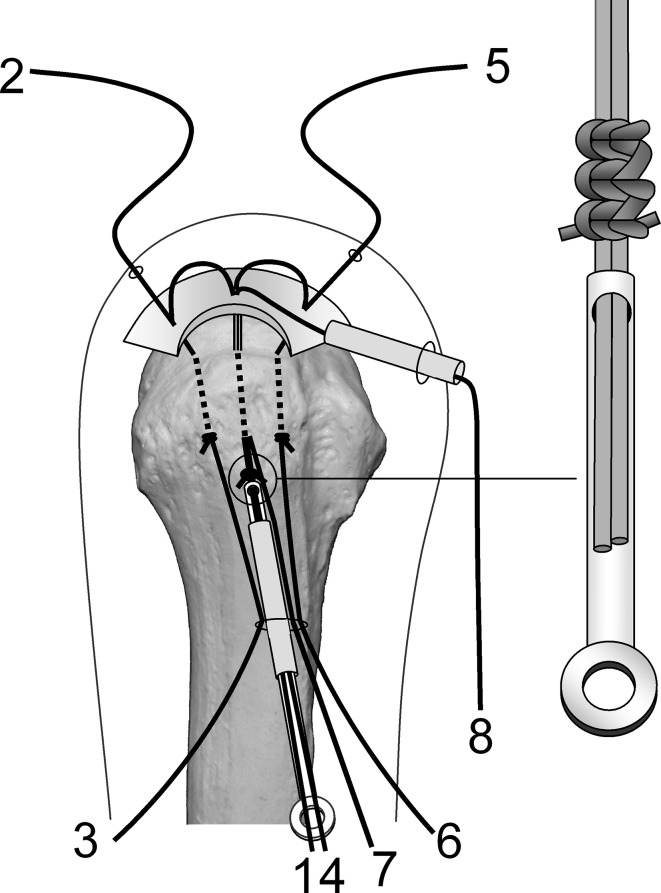
The two polyester limbs (1, 4) that had been pulled out through the lower portal were used as mattress sutures. Subsequently, these were tied with another Number 2 polyester suture using square knots, and this Number 2 suture was twisted around the mattress sutures and ligated by square knotting. This process was repeated again and this knot was next inserted into the greater tuberosity using a knot pusher.

**Fig. 8A–B:**
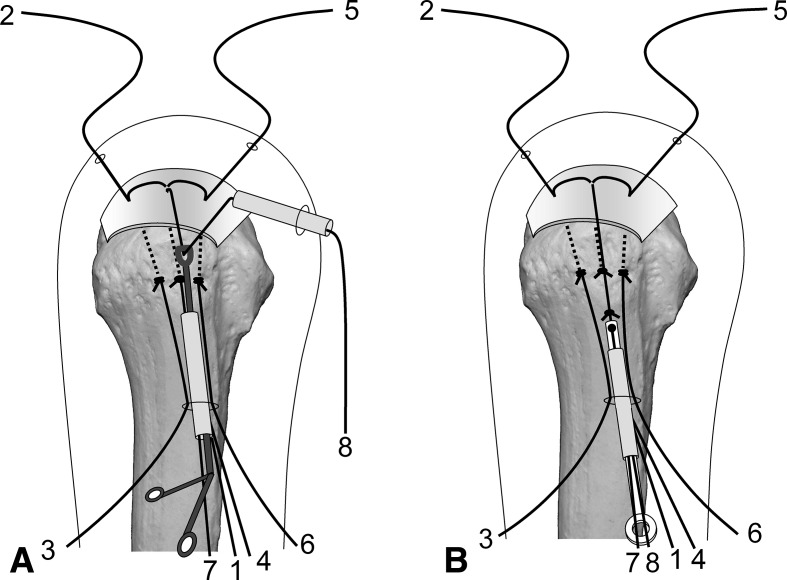
(**A**) One limb of the central polyester bridging suture attached to the rotator cuff (8) was pulled out through the lower portal. (**B**) The limb extracted through the lower portal (8) and the other limb traversing the central bone tunnel (7) were tied in the same manner as the mattress sutures.

**Fig. 9A–C:**
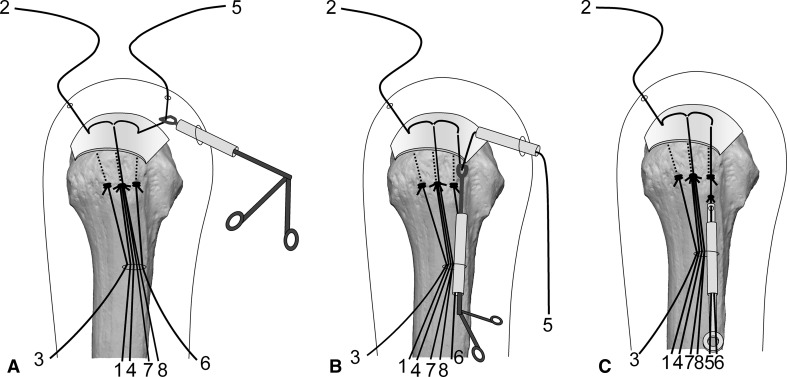
Because the anterior polyester limb (5), which was used as a bridging suture, could not be pulled out directly via the lower portal, it was pulled out through the (**A**) anterolateral and (**B**) lower portals. (**C**) The limb extracted through the lower portal (5) and the other limb passing through the anterior bone tunnel (6) were tied in the same manner as the mattress sutures. For the posterior bridging suture (2, 3), the same process was repeated.

**Fig. 10A–C:**
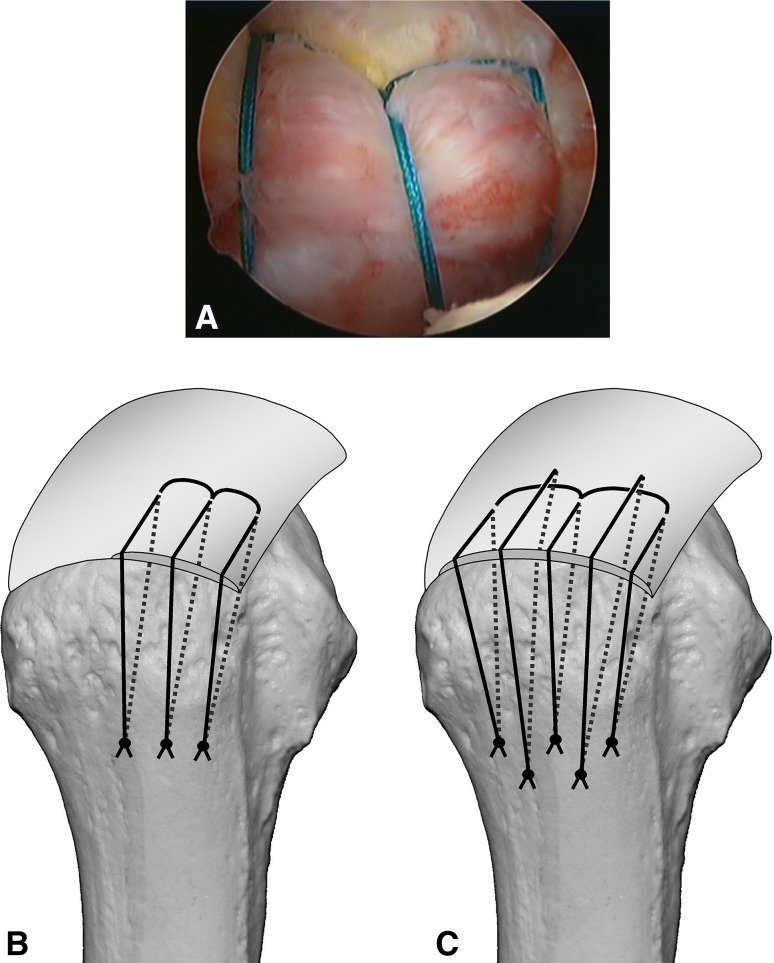
(**A**) An intraoperative view and (**B**) a diagram shows the rotator cuff securely fixed to the footprint with two mattress sutures and three bridging sutures. (**C**) When the AP diameter of the rotator cuff tear exceeded 3 cm, we added two bridging sutures.

Surgery is performed under indirect arm traction (traction weight, 2 kg), with the patient in the lateral position. Five portals are used: anterior, anterolateral, lateral, posterolateral, and lower. The posterolateral one is the main viewing portal; the lateral portal also is used occasionally as a viewing portal. After subacromial decompression and adequate footprint decortication, the following steps are performed (Videos 1 and 2; supplemental materials are available with the online version of CORR^®^). (1) The aiming tip of a customized drill guide is placed on the medial edge of the footprint and three K-wires (2 mm) with perforated tips are inserted into the greater tuberosity at a superiorly directed angle of 55° (Fig. 1A). (2) The stump of the rotator cuff is pulled laterally with a grasper passed through the anterolateral portal and K-wires are threaded through the rotator cuff and skin (Fig. 1B). (3) A nylon suture connected to the center of a 135-cm Number 2 polyester suture is passed through the perforated tip of the anterior and posterior K-wires, after which nylon loops connected doubly in series are passed through the central K-wire (Fig. 2A). Thereafter, these are pulled out through the lower portal, passing through the rotator cuff and the bone tunnels through the greater tuberosity (Fig. 2B). (4) The anterior and posterior polyester suture loops, which have been pulled out, are knotted, after which one end of each loop is cut and pulled superiorly. The knots are sited at the inferior margin of the greater tuberosity (Fig. 3). (5) The central nylon loops and one limb of each of the anterior and posterior polyester sutures that have been inserted through the rotator cuff then are pulled out through the anterolateral portal (Fig. 4). Thereafter, two polyester limbs extracted through the anterolateral portal (Fig. 5) and a 65-cm Number 2 polyester suture for central bridging (Fig. 6) are pulled separately through the lower portal passing through the central bone tunnel, using the nylon loop. (6) The two limbs of the polyester sutures pulled out through the lower portal are used as mattress sutures. These are tied three times with another Number 2 polyester suture using square knots. The knot then is inserted into the greater tuberosity with a knot pusher (Fig. 7). (7) Next, one limb of the central bridging suture attached to the rotator cuff is pulled out through the lower portal (Fig. 8A). (8) The limb of the central bridging suture, which has been drawn out through the lower portal, is tied to the other limb traversing the central bone tunnel in the same manner as the mattress sutures (Fig. 8B). (9) Because the anterior and posterior limbs bound to the bridging suture cannot be pulled out directly through the lower portal, they are pulled out via the anterolateral portal (Fig. 9A) and then to the lower portal (Fig. 9B); thereafter, they are similarly tied (Fig. 9C). (10) The mattress and bridging sutures are further tightened and secured by three half-hitch knots. The rotator cuff is repaired using two mattress and three bridging sutures (Fig. 10A–B). Extra mattress and bridging sutures may be added easily by placing the aiming tip of the drill guide on the rotator cuff after suturing. When the AP diameter of the rotator cuff tear exceeds 3 cm, two extra bridging sutures are added (Fig. 10C).

Patients are asked to use a soft brace that holds the arm in 45°flexion and 45° internal rotation for 4 weeks. Passive elevation is started 2 weeks after surgery by a physiotherapist. When the physiotherapist starts passive elevation, the patient is asked to be careful not to actively elevate the affected arm. Active elevation is permitted at 4 weeks and the patients are permitted to drive 6 weeks after surgery. In shoulders with tears greater than 3 cm, the duration of postoperative fixation and start times of passive elevation, active elevation, and driving are each extended by 2 weeks.

## Patients and Methods

**Fig. 11:**
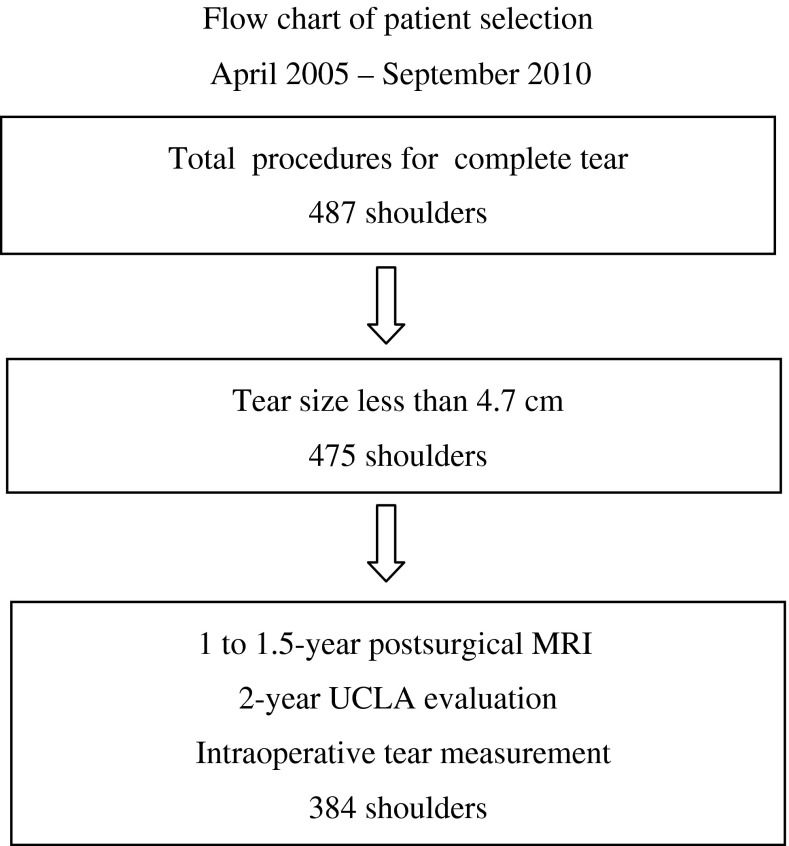
The flow chart shows patient selection from April 2005 to September 2010.

Between April 2005 and September 2010, we performed operations on 483 patients (487 procedures) for complete rotator cuff tears. Four hundred seventy-five (97%) of the procedures were done using our described technique. The indications for performing the technique were: (1) complete rotator cuff tear, and (2) tear less than 5 cm in medial-to-lateral diameter. The contraindications to this technique were: (1) global tear greater 5 cm in medial-to-lateral diameter, and (2) cases where the stump of the torn rotator cuff did not emerge across the top of the humeral head under traction. Criteria for inclusion in this study were: (1) UCLA assessment 2 years after surgery; (2) MRI performed 1 to 1.5 years after surgery; and (3) complete surgical records including accurate intraoperative measurement of the size of the rotator cuff tear. Exclusion criteria were; (1) previous fracture; (2) revision rotator cuff repair; and (3) preoperative cervical radiculopathy or axillary nerve palsy. This resulted in a study sample of 380 patients (384 shoulders [right, 258; left, 126; women, 174; men, 206], 81% of the procedures performed using this technique during the period in question (Fig. 11). In 262 patients (264 shoulders) who underwent surgery in January 2008 or later, EMGs were performed before surgery and 1 month after surgery. Patient age at the time of surgery averaged 67 years (range, 35–86 years). The medial-to-lateral diameter of tears was less than 1 cm in 95 patients (96 shoulders), 1 to 3 cm in 216 patients (219 shoulders), and 3 to 4.7 cm in 69 patients (69 shoulders). This procedure was performed by three different surgeons (NI, RM, SO). Minimum followup was 2 years (mean, 3.3 years; range, 2–7 years). The 91 excluded patients (91 shoulders) either did not have a 1-year postsurgical MRI, 2-year UCLA evaluation, intraoperative tear measurement, or were the cases corresponding to the excluding criteria or lost to followup.

**Fig. 12:**
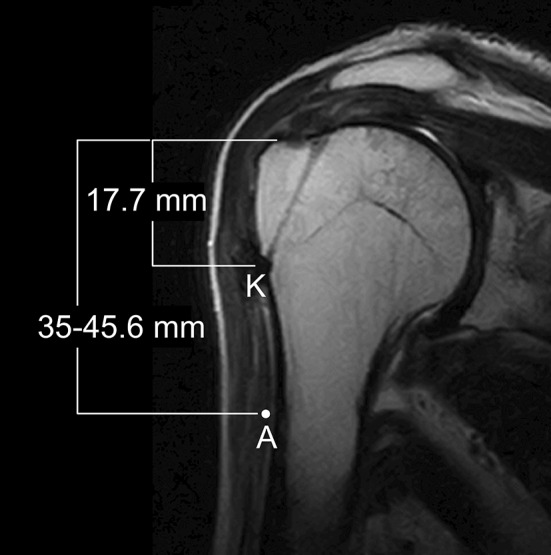
A postoperative MR image shows the average distance from the superior border of the greater tuberosity to the insertion point of the K-wires (K) as 17.7 mm. The distance between the superior border of the greater tuberosity and the axillary nerve (A) has been reported as 35 to 45.6 mm.

One to 1.5 years after surgery, MRI of the suture site on the rotator cuff was used to evaluate the tears, which were classified according to the system of Sugaya et al. [19]. Sugaya Type IV denotes the presence of a minor discontinuity in oblique coronal and oblique sagittal views of T2-weighted images and suggests a small tear. Type V denotes the presence of a major discontinuity and suggests a medium or large tear. Before and 2 years after surgery, we evaluated the shoulders using total scores on the UCLA Shoulder Rating Scale [6], in which 35 is the best possible score. To evaluate axillary nerve damage, EMGs of the deltoid muscle were performed before and 1 month after surgery. Moreover, to check for axillary nerve safety, the distance from the superior border of the greater tuberosity to the point of insertion of the K-wires was measured on postoperative MR images (Fig. 12). We watched carefully for any problems with the bone tunnels throughout surgery.

## Results

Retears were uncommon in this series. Using MR images for screening, we identified Sugaya Type IV and V tears in 24 patients (24 shoulders) (6%); these were considered retears. Based on the size of the original tear, the incidence of retear was 4% (4/96) for small tears (< 1 cm), 5% (11/219) for medium tears (1–3 cm), and 13% (9/69) for large tears (3–4.7 cm) (Table 1). Among the 26 patients (26 tears) with tears larger than 4 cm, retears were recognized in six patients (six shoulders) (23%). Using the same technique, we performed repeat rotator cuff surgery in four of these 24 patients with retears.

**Table 1:** Retear rate according to tear size

Tear size	< 1 cm (n = 96)	1–3 cm (n = 219)	3–4.7 cm (n = 69)	Overall (n = 384)
Number of shoulders with retears	4	11	9	24
Retear rate (%)	4	5	13	6%

The preoperative mean UCLA total shoulder score was 19.1 (range, 5–30) and the postoperative score 32.7 (range, 15–35) (Table 2). Postoperative results were excellent (34–35) in 55%, good (29–33) in 33%, and poor (< 29) in 12%. The mean preoperative UCLA score was 21.6 and postoperative score 34.3 for small tears. They were 20.2 and 33.5 for medium tears (1–3 cm), and 12.1 and 28 for large tears (> 3 cm), respectively. The mean operating time was 80 minutes for small tears, 95 minutes for moderate tears, and 176 minutes for large tears.

**Table 2:** Scores on the UCLA Shoulder Rating Scale (n = 384 shoulders)

Time of evaluation	UCLA score (points)					
	Pain	Function	Forward flexion	Muscle strength	Satisfaction	Total
Preoperative	3.8	7.6	3.9	3.7		19.1
Postoperative	9.1	9.5	4.7	4.7	4.6	32.7

No damage to the axillary nerve was detected by postoperative EMG in any patient. The distance between the top of the greater tuberosity and the insertion point of the K-wire on postoperative MR images (Fig. 12) averaged 17.7 mm (range, 6–34 mm).

A bone tunnel-related issue was encountered in only one shoulder during the procedure. This issue was breakage of the bone tunnel by the suture having been passed through it. No other damages of the bone tunnel were observed on postoperative radiographs, CT scans, and MR images.

## Discussion

Currently, arthroscopic rotator cuff repair is the most widely used technique for managing rotator cuff tears. Therefore, improvement of arthroscopic cuff repair is a major topic. Because anchors are used in almost all arthroscopic rotator cuff repairs, various anchor-related shortcomings remain with this technique, such as difficulty with revision surgery because of the presence of anchors in the greater tuberosity, anchor dislodgement, and knot impingement. We have developed a more economical technique in which the only extra cost involved is that of the suture materials, and problems related to anchors are eliminated. In this study, we describe the details of this procedure and report its outcomes in terms of (1) retear rate, (2) total score on the UCLA Shoulder Rating Scale [6], (3) axillary nerve preservation, and (4) issues concerning bone tunnels.

In our series, the overall postoperative retear rate was 6% using this technique. This rate is low compared with rates reported for suture-anchor-based methods [2, 10, 14, 20]. In this context, it should be noted that there are no global tears (medial-to-lateral diameter of torn rotator cuff greater than 5 cm) in our series because we use an open multiple muscle transfer technique for such tears. Because retear rates are proportional to tear size in other studies [10, 20] and were in our series, exclusion of patients with such large tears could explain our low retear rate. Therefore, it is meaningless to simply compare the overall retear rates in our series with those reported by others whose series had a different distribution of tear size. Nevertheless, the retear rate in our series was low when compared with those reported by others for small and medium tears [10, 20]. This relatively low retear rate is a potential advantage of our procedure, in which the rotator cuff is pulled peripherally by mattress sutures and compressed tightly to the footprint by bridging sutures.

Two years after our procedure, the mean UCLA total shoulder score was 32.7 points. This is comparable to other series that have reported this end point at a minimum of 2 years after suture-anchor repairs [5, 7, 10, 11].

Protection of the axillary nerve is critically important in this procedure, in which K-wires are inserted through the inferior margin of the greater tuberosity. Postoperative MR images showed that the mean distance between the top of the greater tuberosity and the insertion point of the K-wires was 17.7 mm. Given that the mean distance between the upper margin of the greater tuberosity and the axillary nerve is variously reported as 35 mm [8] and 45.6 mm [15] (Fig. 12), the axillary nerve should be relatively safe from injury from K-wires in this procedure. We confirmed the absence of axillary nerve palsy by clinical findings and postoperative EMGs.

Problems with bone tunnels are a theoretical and perhaps an actual concern with transosseous techniques. Sixteen years ago, a cadaver study showed that transosseous rotator cuff suturing was inferior to suture-anchor repairs as a result of cyclic loading [4]. However, the transosseous suturing used in that study was simple suturing of the rotator cuff using a short bone tunnel in an area of weak bone cortex on the lateral margin of the footprint. We believe that the approach studied by Burkhart et al. [4] is substantially different from our technique, which combined a long bone tunnel extending from the inferior margin of the greater tuberosity to the medial edge of the footprint with two sets of mattress and three bridging sutures. Therefore, we believe that the conclusions of the cadaver study [4] do not apply to our technique. In our study, breakage of the bone tunnel was identified during the procedure in only one patient; this patient had severe osteoporosis that was apparent on plain radiographs of the lumbar spine.

Our study does have several limitations. Because we did not have a cohort for comparison, we compared our UCLA shoulder scores and retear rates with data from other published studies. The second limitation is that we did not perform EMG on all shoulders. However, we examined all shoulders clinically monthly for the first postoperative year and again at a minimum of 2 years. We identified no axillary nerve palsies. In addition, we assessed postoperative tendon integrity by MRI at a minimum of 1 year (mean, 13 months), whereas we performed clinical assessments 2 years after surgery. We believe this limitation is at least partially offset by our consistent approach to assessment of tendon integrity, which involved postoperative MRI in all patients. Although 19% of patients who underwent this procedure were lost to followup, we believe the followup rate of 81% was sufficient to accurately evaluate the procedure. Because we performed this technique for all complete rotator cuff tears except for global tears (> 5 cm in medial-to-lateral diameter), there was no selection bias. There also is a limitation related to the surgical technique. Our method cannot be used in shoulders where the stump of the torn rotator cuff does not emerge across the top of the humeral head under traction. By expanding decortication of the footprint to the inner side, large rotator cuff tears also can be repaired. However, as shoulders with tears larger than 4 cm had a retear rate of 23%, we do not recommend this technique in such cases.

With our approach, we found a low retear rate, excellent shoulder scores, no nerve injuries, and minimal problems related to bone tunnels. It also allowed us to avoid the use of suture anchors, which can dislodge and add cost to the procedure. In rotator cuff repairs, transosseous sutures may generate a greater bonding force than anchor sutures [17]. Because suturing restricts rotator cuff movement [1], transosseous suturing is advantageous in rotator cuff repair. In our procedure, mattress sutures are required to draw the cuff stump peripherally and apply adequate initial fixing power to the footprint. Because the K-wires are threaded diagonally through the rotator cuff, strong traction is exerted by the sutures on the rotator cuff.

The presence of anchors in the greater tuberosity makes reoperation difficult. These disadvantages are especially obvious when numerous metal anchors have been used. While probably preferable, absorbable anchors still have the disadvantage of requiring drill holes in the bone. Although revision arthroscopic rotator cuff repair using anchors has been reported [9, 12, 13, 18], the authors’ only reference to previously placed anchors is as follows: “they are removed only when prominent or crowding of the greater tuberosity is seen” [12]. In many cases in which anchor suturing is used, several anchors are inserted into the greater tuberosity, making it difficult to place additional anchors or create bone tunnels for transosseous suture repair. The situation worsens when additional anchors are inserted and second retears occur. Because our anchorless technique uses only polyester sutures, it offers the added advantage of facilitating revision surgery. To obtain satisfactory initial fixing power without applying excessive tension to the rotator cuff, the use of absorbable mattress sutures should be considered. Thus, a comparison study of nonabsorbable and absorbable sutures is required. It is our intention to pursue this issue.

## Electronic supplementary material

Below is the link to the electronic supplementary material.

Supplementary material 1 (MPG 48700 kb)

Supplementary material 2 (MPG 42624 kb)
